# Sleep Latency and Physical Activity in Older Adults with Mild Cognitive Impairment: Oura Ring Longitudinal Study

**DOI:** 10.1093/geroni/igaf122.4193

**Published:** 2025-12-31

**Authors:** Jungjoo Lee, Junhyoung Kim, Kangeun Lee, Aubrey Carter

**Affiliations:** Texas A&M University, Texas A&M University, Texas, United States; Texas A&M University, College Station, Texas, United States; Indiana University, College Station, Texas, United States; Texas A&M University, College Station, Texas, United States

## Abstract

**Background:**

Older adults with mild cognitive impairment (MCI) experience delayed sleep latency due to neuropsychological imbalance. While physical activity may enhance sleep quality, reliance on self-reported measures can reduce reliability because of recall bias.

**Aim:**

To investigate the longitudinal relationship between sleep latency and physical activity intensity in older adults with MCI through continuous monitoring using a smart ring system.

**Method:**

Seven older adults with MCI wore Oura smart rings for 14 days. Equipped with photoplethysmography, accelerometer, and gyroscope sensors, the rings calculated sleep latency as the interval from attempting sleep to the first light sleep episode, determined by reduced movement and thermoregulatory shifts. Physical activity intensity was estimated from heart rate variability and skin temperature, and classified as light, moderate, or vigorous based on the Metabolic Equivalent of Task. Data were analyzed using generalized estimating equations.

**Results:**

Vigorous activity significantly reduced sleep latency. Each additional second of vigorous activity corresponded to a 0.60 second decrease in latency (B = -0.60, SD = 0.21, p < .01, CI [-1.02, -0.18]). Moderate activity showed no significant association (B = 0.04, SD = 0.11, 95% CI [-0.17, 0.26]). Light activity showed a near significant reduction of 0.02 seconds per second (B = -0.02, SD = 0.01, 95% CI [-0.05, 0.01]).

**Conclusion:**

Only vigorous physical activity was significantly associated with reduced sleep latency. Light activity showed a trend toward improvement, while moderate activity had no clear effect. These results suggest that intensity specific interventions may be required to improve sleep in this population.

